# Revisiting the child health-wealth nexus

**DOI:** 10.1186/s13561-016-0120-8

**Published:** 2016-08-30

**Authors:** Adnan M. S. Fakir

**Affiliations:** BRAC University, Dhaka, Bangladesh

**Keywords:** Child health, Endogeneity, India, Anthropometry, Instrument variables

## Abstract

**Objective:**

The causal link between a household’s economic standing and child health is known to suffer from endogeneity. While past studies have exemplified the causal link to be small, albeit statistically significant, this paper aims to estimate the causal effect to investigate whether the effect of income after controlling for the endogeneity remains small in the long run. By correcting for the bias, and knowing the bias direction, one can also infer about the underlying backward effect.

**Design and setting:**

This paper uses an instrument variables two-stage-least-squares estimation on the Young Lives 2009 cross-sectional dataset from Andhra Pradesh, India, to understand the aforementioned relationship.

**Results:**

The selected measure of household economic standing differentially affects the estimation. There is significant positive effect of both short-run household expenditure and long-run household wealth on child stunting, with the latter having a larger impact. The backward link running from child health to household income is likely an inverse association in our sample with lower child health inducing higher earnings. While higher average community education improved child health, increased community entertainment expenditure is found to have a negative effect.

**Conclusion:**

While policies catered towards improving household wealth will decrease child stunting in the long run, maternal education and the community play an equally reinforcing role in improving child health and are perhaps faster routes to achieving the goal of better child health in the short run.

## Background

Over the past few decades, while many South Asian countries have enjoyed economic improvements, a reflection of such has not been seen in terms of child nutrition [1–3]. Mild to moderate malnutrition has been recognized to account for more than half of the 10 million children dying each year from preventable diseases [4]. Malnutrition causes a child to be more vulnerable to infectious diseases with several identified long term impacts. Delayed cognitive development [5], poor educational attainment [6] and lower intellectual and physical abilities in adult life leading to lower lifetime earnings [7] have been all linked to deprived nutritional status.

As a broad function, malnutrition is determined by health inputs, the local health environment and the child’s genetic endowment [8]. Health inputs range from nutrient intake, level of care, quality of medical services, sanitation and toilet facilities and drinking water purity, many of which are correlated with income. Thus the ‘income effect’ can be defined as a summation of partial effects of the health inputs on a child’s health status. Given the multitude of pathways through which income can establish its relationship with child status, it is important to understand their mechanisms before modelling child health.

While a rise in household income can improve child health, there is an acknowledged backward effect where lower child health can motivate parents to work harder to earn more income for treatment. This simultaneity raises an issue of endogeneity, which unless accounted for, will lead to biased results. This essay scrutinizes the role of income on a child’s growth using instrument variables estimation in order to tackle the underlying endogeneity.

The data used to perform the analyses comes from the third round of the Young Lives study conducted in Andhra Pradesh, India in 2009. Even with the impressive growth of roughly 8 % in India during this time, malnutrition rates remain high making it particularly relevant to identify the role played by income on child’s health. Child malnutrition in India is thought to be responsible for nearly half of global child deaths, with almost 48 % of the children under the age of 5 being stunted in India [9] making this a major policy question that needs to be adequately addressed. In particular, I wish to identify significant causal effects, because if household economic standing does not substantially improve child health, then policy interventions set to increase income will not necessarily improve child health.

The objectives of this essay can be summarized as follows: (i) estimate the impact of income on child health, accounting for potential endogeneity while controlling for individual, household and community factors; (ii) using consumption expenditure and wealth index as proxies of temporary and permanent effects of household economic standing and understanding their differential effects; and (iii) investigation of community level factors, that also play pivotal roles in understanding child health, often overlooked in the literature. For the latter, I emphasize particularly on community level education and entertainment expenses, which can contribute up to one-third of household expenditure in rural India, as indicated by Banerjee and Duflo [10].

## Child health

### The problems of income effect estimation

Height-for-age, or stunting, is used in this analysis as an indicator for long-term health. The case for height-for-age, as a marker for long-term health outcome is supported in several studies [11–13] which assert an association between taller height and better health, better cognitive outcomes, greater earning ability, and higher probability of being employed.

The causal effect of income on child health has been probed by several authors [8, 14]. Unarguably a large portion of income’s role on child health operates through other factors, most notably the health inputs. Food intake, household sanitation, quality of medical care received that directly affect child health, are all correlated with income [15]; in other words, the estimated income coefficient reflects a large portion of the effect of the health inputs on child health. Income, on the other hand, also depends on level of education [16]. Thus the estimated income coefficient also captures an ‘income effect’ portion of the ‘total education effect’ [17]. One should ideally control for these factors to better understand the true role of income, which raises an alarming question as to whether the causal relationship is actually a misinterpreted correlation.

Pal [18] points out that the instrument of choice used to measure income is crucial in analysis as estimates often differ based on the variable of choice. Per capita income, consumption expenditure and wealth indexes all provide, to various extents, measures of the socioeconomic status and resource availability of the household, each with their advantages and problems. While permanent income and wealth status tend to be better estimates of resource availability, they are often difficult and expensive to measure [19]. Hence even though current income tends to have transitory components it is often used as a proxy.

Thomas, Strauss and Henriques [17] further point out measurement problems related to current income. Namely (i) respondents may be unwilling to disclose their income, (ii) income from self-employment is hard to measure and (iii) if only the mother of the household is surveyed, she may not know the total income of the household. While the Young Lives 2009 dataset allow us to compare consumption expenditure with a generated wealth index, it is likely that the measure of consumption expenditure also face similar problems. This measurement bias can be another source of endogeneity further stressing the use of appropriate estimation methods.

As mentioned, the backward effect of income and child health stems from parents with children who are ill, deciding to work longer hours to pay for medicine, thus earning more income. Since such negative shocks could increase household income, the impact of income on child health would be underestimated. However, parents might also work fewer hours due to child’s illness, in order to spend more time with the child, thereby earning lower income, where its impact would be overestimated [8]. Thus care should be taken when exploring the direction of potential bias.

Janet Currie’s [20] paper is a comprehensive survey exploring the aforementioned dichotomy of cause and effect between household economic status and child health. To illustrate both sides of the debate, Berger, Paxson and Waldfoget [21] in a study on US children conclude that although income does have significant effect on child health, it is small compared to maternal education or maternal health. On the contrary, the effect of income on child health has not always been found as significant. Using a two round panel Vietnam Living Standards Survey (VLSS) from 1993 and 1998 and instrument variables to account for endogeneity, Glewwe, Koch and Nguyen [8] note the role of growth in household income on child health to be small and questionable. However, Alderman, Hoogeveen and Rossi [22] in another longitudinal study in Tanzania also instrument per capita consumption and find definitive positive impact of income on child health, acting in association with nutrient uptake.

Another argument for the income effect on child health operate through the fetal origins hypothesis as postulated by Barker [23] (see Gluckman and Hanson [24] for a survey literature). Several recent studies have shifted the attribution of lower child weight at birth to environmental and maternal factors caused due to lower economic standing, as opposed to “inferior” genetic endowments. Barker [23] postulates that insufficient nutrition, due to shocks or lower economic standing, *in-utero* hampers fetal growth, eventually leading to increased disease prevalence. A large and recent body of research has focused on long-term adult health outcomes from exposure to intrauterine shocks during wars, famines and recessions, finding that those in-uetro during such events suffer from lower long term health [12, 25–28].

Løken, Mogstad and Wiswall [29] find an increasing, concave relationship between household income and children’s outcomes using nonlinear IV estimators. On the other hand, Cameron and Williams [30] look into whether the effect of low income on child health differs with increasing child age, as would be suggested if the effect accumulates over time, finding no such effect in Indonesia.

### The control factors

The role of education in relation to child health has been closely scrutinized. Smith and Haddad [31] report that almost half the reduction in underweight children between 1970 and 1995 is explained by increases in female adult literacy. In societies where mothers are the main caregivers of the child, maternal education has been shown to have a stronger and significant effect on child health than paternal education on numerous occasions [32–34].

However, Behrman and Rosenzweig [35] mention that many of the large-scale studies showing a link between maternal education and child nutrition are not as informative because they do not set up controls for inter-generationally correlated genetic endowments, leading to endogeneity problems. Since taller parents are more likely to have children with better health endowments, one way of controlling for this is simply by using the heights of parents [36].

Studies have also found a strong correlation between maternal education and indicators of care, such as better sanitation practices, better child feeding, timely immunization etc.[37, 38]. Cebu Study Team [39] used a Philippines longitudinal data to find that educated mothers are better at recognizing threats to the health of their children. The causal link is that mother’s education induces behavioural changes which lead to better child health. Fakir and Khan [40] similarly report that after controlling for health knowledge and health-seeking behaviour, in an urban slum in Bangladesh, the effect of maternal education on child health is no longer significant.

In an interesting cross-country study, Desai and Alva [38] find that including regional and community fixed effects lowers the impact of education. They conclude that mother’s education level thus acts as a proxy for geological area of residence and socioeconomic status, which must be controlled for in order to investigate the role of parental education. In fact, Thomas, Strauss and Henriques [17] show that almost all the impact of maternal education on child survival could be explained by how easily the mothers could access information, such as read newspapers, watch TV and/or listen to radio. Desai and Alva [38] and Frost, Forste and Haas [41] also concluded that maternal education influences health seeking behaviour which in turn influences child health. In fact, Grossman [16] suggests that education plays a larger role on improving child health than household income.

It is also worth noting that there is differential effect of the determinants in regard to child gender and geographical residence. Pal [18] uses a probit model of nutritional status on a 1987-89 dataset from West Bengal to find that female literacy improves the nutritional status of boys at the cost of girls, strongly implying that male and female children follow different health functions. On the other hand, Bourne and Walker [42] using the 1981 Indian census data find that maternal education reduces mortality rates of girls more than boys.

The rural-urban differential of maternal education is often stressed, with a greater impact of income and education in urban areas. While Bicego and Boerma [43] attribute this due to availability of broader social and economic support, Glewwe, Koch and Nguyen [8] and Caldwell [44] suggest that a synergistic interaction between health services and education play a more important role. An example would be from Caldwell [45], who shows that in Nigeria the benefit of maternal education is greater in villages with access to a hospital than those without it.

Studies have also shown that the overall community level of education quite often play a pivotal role in determining child health, especially when the mothers face lower education. Moestue [46] uses the first round of Young Lives dataset to explore this and has found parental education to have a stronger impact on child nutrition where community level maternal literacy is higher. Similarly, Moestue and Huttly [47] find positive impact of community level literacy on Vietnamese children after adjusting for parental education and other cofounders.

This spill-over effect of community level factors is succinctly captured by Moursund and Karvdal [48] where they show that the average educational level of other women in the census-enumeration area has a positive effect on contraceptive use of women. Cleland and Jejeebhoy [49] also report lower fertility rates amongst women with little or no education but living in educated communities, compared to similarly educated women living in communities with lower overall education. Moestue, Huttly, Sarella et al. [50] use a unique dataset on mothers’ networks to show that mothers from poorest household benefit the most from having a more ‘literate’ network in improving child health. Based on these findings, community-level factors are explicitly controlled for while modelling child health, and are the secondary variables of interest in this paper.

Finally, Banerjee and Duflo [10], report that poor households in India spend as much 30 % of their income on entertainment. This study looks into this aspect, as to whether community-level entertainment expenditure, representing high relative leisure expenses, plays any negative role, via negligence, towards child health.

## The instrument variables (IV)

I compare two measures of household economic standing in this study: household consumption expenditure and the wealth index, estimated separately. The idea of using the instrument variables estimation is to purge the household economic standing measures of their correlation with the error that arises due to the endogeneity. The difficulty is the simultaneity of household economic standing with child health; while higher wealth and expenditure may be indicative of improved child health, poor child health requiring medical attention may cause the economic standing of a household to fall due to negative impacts on the parents’ labor supply [51].

Addressing this issue, earlier seminal studies have commonly used unearned income [52] productive assets and demographic composition factors [53] as identifying instruments for household expenditure. There have been arguments however, against these instruments as unearned income may simply reflect past labor supply and unobserved productivity or preferences with child health, while decisions to purchase certain assets or goods may also be jointly determined with child health, rendering these variables endogenous.

Alderman and Garcia [53] acknowledged this and used community averages for these endogenous variables as instruments, arguing that their incidence at the community level will affect an individual household’s decision concerning expenditures. For example, if in a local community a higher percentage of children are vaccinated, it may influence individual households to spend more behind proper vaccination. This nature of peer effect is thus taken advantage of in being used as instruments.

On the other hand Handa [54] used ownership of durables, income from property, access to a telephone, type of residence and material used in house walls as instruments of household expenditure and wealth. Keeping to earlier studies, I use the following two instruments to solve the identification problem: (i) amount of non-irrigated land owned, and (ii) amount spent on telephone rates and mobile cards purchase.

The difficulty in finding appropriate instruments rest with satisfying the exclusion restrictions, relevance and exogeneity assumptions. While the relevance assumption can be tested, whether the instruments are uncorrelated with the error is a matter of economic reasoning. The rationale for selecting the two instruments is as follows:

(i) *Amount of non-irrigated land owned*
  The idea behind the exclusion restriction is that it should not have a direct causal effect on child health but only through household expenditure or wealth. Firstly, land ownership is not included in the construction of our wealth index, and certainly not a part of household expenditure, thereby excluding the possibility of the variable being endogenous. Moreover, by considering the amount of non-irrigated land a household owns which mostly reflects the homestead land and is not tied to any income generation, the instrument explicitly avoids any causal effect running from the variable to child health.
  Thus while it is unlikely that the amount of non-irrigated land owned by a household will affect child health, the exclusion restrictions assumption can be violated if lower child heath caused the household to sell their land. This will cause amount of non-irrigated land owned by the household to be correlated with the error violating the assumption. A simple pair-wise correlation between child health and sold land returns an insignificant association ($p$-value = 0.5137). This association is also found to be positive (albeit insignificant) which goes contrary to our statement above. Furthermore, roughly 8 % of the households in our dataset sold land within the past three years. While there is no data on the reason why these households sold the land, a simple tabulation shows that the average child height-for-age standard deviation for households that sold land was −1.45, compared to average child height-for-age standard deviation for those who did not sell land was −1.50. This indicates that the average child health was better for those who sold land, suggesting that it is unlikely that child health was the reason for selling the land.
  Another such problem would arise if household shocks caused the land to be sold off. By explicitly controlling for household shocks, bringing them out of the error and into our model, the model ensures that the instrument is not correlated with the error.
(ii) *Amount spent on telephone rates and mobile cards purchase*
  The amount of money spent behind verbal communication would reflect a household’s economic standing and social network, while not affecting child health directly. A more well off household would be able to afford to spend more behind verbal communication; similarly if households have more relatives abroad or outside of the locality, from whom they receive money as remittance, they are more likely to spend more behind verbal communication. Thus expectedly, there are significant positive Pearson correlations of 0.1441 ($p$-value = 0.000) between verbal communication and number of relatives living abroad and 0.0336 ($p$-value = 0.0415) between verbal communication and amount of money received from abroad. The lack of a causal effect on child health but high correlation with a household’s economic status makes this an appropriate instrument for our analysis.

## Methods

The health of an individual child *i*, living in community *c* at time *t*, ${H}_{ict}$, can be determined by primarily three types of variables: a vector of observable individual health inputs ${HI}_{ict}$, such as pre-natal care, food nutrient intake, quality of medical care, medicine, household sanitation and toilet facilities, drinking water quality etc.; a vector of the local health environment ${E}_{ct}$, at community level *c* and time *t,* which includes the characteristics of the local community that directly affect the child’s health status such as local disease prevalence, air and water pollution levels etc.; and child’s genetic health endowment, ${\alpha}_i$, inherited form his/her parents:

$$ {H}_{ict} = f\left(H{I}_{ict},\ {E}_{ct},\ {\alpha}_i\right) $$

While both the genetic health endowment and the local health environment is exogenous, it can be argued the latter to be endogenous given that households “migrate to healthier environments or take measures to improve the local health environment” [8]. This health environment variation at the community level can be controlled for using community fixed effects. Next, substitute the health inputs into the child health production function:

$$ {H}_{ict} = f\left({Y}_{ict},\ M{S}_{ict},\ F{S}_{ict},\ {\eta}_i,\ {E}_{ct},\ {\alpha}_i\right) $$

Notably, the health inputs depend on endogenous household economic status, ${Y}_{ict}$. In addition, mother’s schooling ${MS}_{ict}$, and father’s schooling $,\;{FS}_{ict}$, as well as unobservable parental preferences ${\eta}_i,$determine both quantity and quality of health inputs that a child receive.

Given that the study uses stunting, the child’s height-for-age z-score, as an indicator of child’s health status, ${H}_{ict}$, the next step is to transform the reduced form health demand function, which is the structural equation, into the primary equation of interest:

$$ zscor{e}_{ict} = {\beta}_0+{\beta}_1{Y}_{ict}+{\beta}_2M{S}_{ict}+{\beta}_3F{S}_{ict}+{\beta}_4{E}_{ct}+{\beta}_{\left(x+4\right)}{X}_{ict}+{\nu}_{ict} $$

$$ where,\ {\nu}_{ict} = {\eta}_i + {\alpha}_i + {\mu}_{ict} $$

The idiosyncratic error term is represented by ${\mu}_{ict}$ and ${X}_{ict}$ is a vector of exogenous child and household characteristics.

Using the two stage least squares (2SLS) for the instrument variables (IV) estimation, the first stage reduced form equation is:

$$ {Y}_{ict} = {\beta}_0+{\beta}_1M{S}_{ict}+{\beta}_2F{S}_{ict}+{\beta}_3{E}_{ct}+{\beta}_4{Z}_{it}+{\beta}_{\left(x+4\right)}{X}_{ict}+{\nu}_{ict} $$

where ${Z}_{it}$ now represents a vector of the two instruments. We predict the fitted values from the first stage and insert that back into the structural equation, as the IV, replacing the endogenous regressor, before performing ordinary least square (OLS) estimation on the second stage, which is the primary equation of interest.

## Data preparation

The third round of the Young Lives dataset for Andhra Pradesh, India conducted in 2009 is used for this study. Andhra Pradesh is the fifth largest state in India with only 27 % of its population living in urban areas. Details about the fieldwork can be found in Attawell [55]. Random sampling methodology was applied to select study households within a sample of communities located in each site. In Andhra Pradesh, this amounted to 102 communities. Total number of children surveyed was 2,937 in 2009. An attractive depth of data collected and accuracy in recording height and age make the Young Lives dataset an ideal choice for anthropometric health studies.

Finally, the regressions reported under section 05 consist of varying observations, although very slightly, because of missing or flagged height-for-age data, missing community level health facility information, missing mother’s education data or missing consumption expenditure or wealth index information. For comparison, the reported descriptive statistics are for the smallest sample size regression of 2,465 observations that are used in analysis.

Expectedly, a positive association of child health with both per capital consumption expenditure (in hundreds) and the wealth index exists, although the variability is larger in case of the latter. The wealth index is constructed by Young Lives as a weighted average of the following variables: (1) rooms per person, (2) housing floor quality, (3) housing roof quality, (4) ownership of radio, (5) ownership of bicycle, (6) ownership of TV, (7) ownership of motorbike, (8) ownership of motorized vehicle, (9) ownership of telephone, (10) ownership of modern bed or table, (11) access to electricity, (12) access to piped water, (13) access to own pit latrine or flush toilet, and (14) access to cooking fuel.

Table 1 reports the descriptive statistics of the variables used for analysis against child stunting. The reported $P$-values are just for statistical association checks from appropriate ${\chi}^2$and $t$-tests. Under the percentage stunted column, a child is reported to be stunted if the height-for-age z-score is below two standard deviations from normal.

**Table 1:** Descriptive Statistics (*n* = 2,465)

Variable	Sub-Category	Mean	*P*	Percent Stunted	Variable Type
Primary Variables of Interest					
Wealth Index	Quartile 01	0.276		43.1	Continuous
	Quartile 02	0.467		34.9	
	Quartile 03	0.589		28.4	
	Quartile 04	0.745	<0.000	17.3	
Per Capita Consumption Expenditure (in hundreds)	Quartile 01	4.317		36.7	Continuous
	Quartile 02	6.962		35.5	
	Quartile 03	9.539		31.2	
	Quartile 04	15.89	<0.000	24.0	
Child Level Controls					
Child Age in Months		123.8	<0.000	-	Continuous
Sex of Child (1 = female)	Male	0.522		31.7	Dummy
	Female	0.478	0.910	30.1	Dummy
Child in School (1 = yes)		0.916	0.268	-	Dummy
Parental Education Controls					
Mother’s Education	No Education	0.557	<0.000	38.5	Dummy
	Primary	0.150	0.793	30.0	Dummy
	Secondary	0.232	0.244	21.0	Dummy
	Higher	0.060	<0.000	9.15	Dummy
Father’s Education	No Education	0.376	0.072	39.0	Dummy
	Primary	0.174	0.481	32.9	Dummy
	Secondary	0.295	0.168	25.1	Dummy
	Higher	0.156	0.001	19.3	Dummy
Child’s Genetic Endowment Control					
Mother’s Height (in centimetres)		144.11	<0.000	-	Continuous
Household Level Controls					
Number of Adults		3.97	0.860	-	Discrete
Monthly Expenditure on Entertainment		67.13	<0.000	-	Continuous
Anyone smoke in Household (1 = yes)		0.394	0.078	31.3	Dummy
Shock in the Last Three Years (1 = yes)		0.292	0.279	30.9	Dummy
Residence (1 = rural)	Urban	0.277		18.8	Dummy
	Rural	0.723	<0.000	35.6	
Household Religion	Hindu	0.915	0.770	31.8	Dummy
	Muslim	0.073	0.832	19.5	Dummy
	Other Religions	0.012	0.673	31.3	Dummy
Child Ethnicity	Scheduled Castes	0.184	0.389	35.5	Dummy
	Scheduled Tribes	0.123	0.200	42.7	Dummy
	Backward Class	0.477	0.404	31.2	Dummy
	Other Castes	0.214	0.045	19.8	Dummy
Region	Coastal	0.373	0.191	27.2	Dummy
	Rayalaseema	0.271	0.618	31.7	Dummy
	Telangana	0.354	0.069	34.4	Dummy
Community Level Controls					
Public Hospital Available (1 = yes)		0.111	0.098	18.5	Dummy
Private Hospital Available (1 = yes)		0.304	0.012	23.7	Dummy
Public Health Centre Available (1 = yes)		0.209	0.018	27.1	Dummy
Private Health Centre Available (1 = yes)		0.237	0.007	24.0	Dummy
Community Education		3.37	<0.000	-	Continuous
Community Entertainment Expenditure		67.29	<0.000	-	Continuous
Mean Adults in Community		3.97	0.001	-	Continuous

To re-iterate, the Young Lives survey is not country representative and purposefully over-sampled pro-poor areas and then randomly selecting children of the survey age; thus not all siblings from a selected household is present in the data. Overall 31.6 % of our sample is stunted, compared to a national statistics of 48 % in all of India [9], with the average child height-for-age z-score being -1.50.

On average, female children are better nourished than male children in our sample from Andhra Pradesh, which is in contrast to the overall national scenario in India suffering from male children preference [56] and there is a there is decrease in stunting with increasing maternal and paternal education, with a stronger association with that of the mother. A strong association of child’s health with mother’s height is also noted, implying the existence of genetic health transfer. Older children have lower health outcomes where the Pearson’s correlation coefficient yields a significant −0.0918. While a positive Pearson’s correlation coefficient of 0.0034 is found between child health and number of household adults, it is not a significant association. Expectedly, having a smoker in the household relays a significant negative association with child health.

The household “shock in the last three years” dummy has been constructed from positive responses to household theft, death of livestock, drought, flooding, erosions, pests damaging crops, damage of home due to fire, illness or death of the household head and separation of parents, in the last three years, that expressively affected the household. While the ${\chi}^2$ test does not indicate a statistically strong association between child health and household shock, the Pearson’s correlation coefficient yield a significant −0.1148.

We also include region dummies of “Rayalaseema” and “Telangana” to control for the regional heterogeneity, keeping “Coastal” regions as our reference group. Because India is historically caste based, in order to control for such caste based heterogeneity, the children are also grouped into “Scheduled Castes” representing the historically disadvantaged indigenous people and “Backward Class” representing the children from traditionally lower castes, with “Other Castes” as the reference group representing the more privileged caste. Urban children are on average less malnourished than rural children while Muslim children compared to children of other religions, and children born into upper castes compared to children born into scheduled and backward castes, have better child health.

Student’s $t$-tests of having a community level hospital or health centre show significant ($p$-value < 0.000) difference of better child health (both private and public). Under the premise of an overall better educated community positively affecting child health, the “community education” variable was constructed as the average of mothers’ education in a community. Similarly, “community entertainment expenditure” and “mean adults in community” variables were constructed as averages of the respective variable within a community. For each of the three variables, significant association with child health is noted. The idea behind including the “community entertainment expenditure” is to understand whether high expenses behind entertainment for low income households, roughly 18 % of per capita consumption expenditure on average for our sample, play any negative role, via negligence, towards child health.

## Results and discussion

The objective of the estimation is to isolate the effect of household’s economic standing on child health, controlling for other determinants, while purging the effect from endogeneity due to simultaneity. Two available measures of household economic standing are used to achieve this - a short run estimation using per capita consumption expenditure and a long run estimation using wealth index. The possible exogeneity of the two measures of economic standing is tested using the Hausman-Wu endogeneity test, rejecting the possibility.

Simultaneity causes the OLS estimator to pick up both forward and backward effects, biasing the estimation. Should poor child health have a positive impact on household income, due to the added incentive to earn more for pursuing treatment, the OLS will underestimate the true effect. On the contrary, should poor child health have a negative impact on household income, due to parents spending more time with the child forgoing earnings from income [8], the OLS will overestimate the true effect.

Six regression models are presented in Table 2, three for each of the measures of economic standing. The OLS results are presented for comparison in columns (1) and (4) for per capita consumption expenditure and wealth index respectively. Columns (2) and (5) conduct 2SLS regressions using the aforementioned instrument variables, which should be robust to the simultaneity bias. Finally, columns (3) and (6) add in the community level variables to the 2SLS estimations. The results are presented in Table 2 including several IV diagnostics.

**Table 2:** Regression Results

	(1)	(2)	(3)	(4)	(5)	(6)
Variable	OLS Expen.	2SLS Expen.	2SLS Expen. with Comm. Variables	OLS WI	2SLS WI	2SLS WI with Comm. Variables
Per Capita Consumption Expenditure (in hundreds)	0.0175***	0.0440**	0.0410**			
	(0.00426)	(0.0173)	(0.0171)			
Wealth Index				0.858***	3.000***	2.940***
				(0.166)	(0.927)	(0.998)
Child Age in Months	−0.00259***	−0.00322***	−0.00326***	−0.00240***	−0.00296***	−0.00297***
	(0.000509)	(0.000676)	(0.000702)	(0.000502)	(0.000592)	(0.000625)
Sex of Child (1 = female)	0.0248	0.0164	0.0212	0.0411	0.0581	0.0591
	(0.0496)	(0.0484)	(0.0502)	(0.0479)	(0.0501)	(0.0524)
Child in School (1 = yes)	0.00603	0.00596	0.00854**	0.00683**	0.00751**	0.00916***
	(0.00367)	(0.00367)	(0.00383)	(0.00345)	(0.00306)	(0.00343)
Mother’s Education = Primary	0.0964	0.0804	0.0663	0.0942	0.0484	0.0239
	(0.0678)	(0.0680)	(0.0729)	(0.0671)	(0.0778)	(0.0862)
Mother’s Education = Secondary	0.132*	0.110	0.0880	0.103	−0.0124	−0.0134
	(0.0672)	(0.0668)	(0.0736)	(0.0630)	(0.0814)	(0.0886)
Mother’s Education = Higher	0.422***	0.339**	0.296*	0.457***	0.311**	0.297*
	(0.139)	(0.147)	(0.156)	(0.127)	(0.145)	(0.157)
Father’s Education = Primary	0.110	0.102	0.0924	0.0904	0.0305	0.0390
	(0.0664)	(0.0648)	(0.0669)	(0.0653)	(0.0721)	(0.0740)
Father’s Education = Secondary	0.106*	0.0851	0.0776	0.0913	0.00313	−0.00127
	(0.0554)	(0.0551)	(0.0561)	(0.0568)	(0.0689)	(0.0698)
Father’s Education = Higher	0.128*	0.0624	0.0492	0.0745	−0.155	−0.164
	(0.0739)	(0.0776)	(0.0786)	(0.0783)	(0.119)	(0.122)
Mother’s Height (in centimetres)	0.00191***	0.00188***	0.00194***	0.00193***	0.00205***	0.00212***
	(0.000428)	(0.000431)	(0.000451)	(0.000431)	(0.000439)	(0.000441)
Number of Adults	0.00565	0.00860	0.0160*	0.00169	−0.00385	−0.000914
	(0.00957)	(0.00970)	(0.00935)	(0.00901)	(0.00994)	(0.0101)
Monthly Expenditure on Entertainment	0.000424*	9.72e-05	0.000133	0.000381**	−0.000293	−0.000106
	(0.000221)	(0.000312)	(0.000307)	(0.000175)	(0.000407)	(0.000308)
Anyone smoke in Household (1 = yes)	−0.159***	−0.163***	−0.137***	−0.123***	−0.0374	−0.0255
	(0.0430)	(0.0417)	(0.0428)	(0.0427)	(0.0531)	(0.0554)
Shock in the Last Three Years (1 = yes)	−0.147***	−0.159***	−0.162***	−0.101**	−0.0514	−0.0622
	(0.0510)	(0.0517)	(0.0535)	(0.0486)	(0.0546)	(0.0558)
Residence (1 = rural)	−0.112**	−0.113**	−0.00769	−0.0640	0.166	0.0902
	(0.0495)	(0.0484)	(0.0941)	(0.0614)	(0.129)	(0.152)
Muslim	0.0863	0.124	0.104	0.0414	0.0673	0.0863
	(0.0800)	(0.0858)	(0.0916)	(0.0854)	(0.0864)	(0.0848)
Other Religions	−0.0548	−0.0443	−0.0569	−0.0183	0.00734	0.0300
	(0.167)	(0.169)	(0.163)	(0.162)	(0.188)	(0.177)
Scheduled Castes	0.262***	0.251***	0.287***	0.201**	0.0528	0.0916
	(0.0981)	(0.0960)	(0.104)	(0.0910)	(0.108)	(0.117)
Backward Class	0.244***	0.199**	0.260***	0.177**	−0.0678	−0.00747
	(0.0806)	(0.0850)	(0.0908)	(0.0759)	(0.133)	(0.146)
Other Castes	0.383***	0.310***	0.360***	0.301***	0.0103	0.0562
	(0.0959)	(0.105)	(0.111)	(0.0895)	(0.155)	(0.171)
Rayalaseema	−0.215***	−0.259***	−0.222***	−0.167**	−0.136*	−0.0857
	(0.0712)	(0.0774)	(0.0838)	(0.0725)	(0.0785)	(0.0780)
Telangana	−0.235***	−0.314***	−0.268***	−0.174***	−0.162**	−0.0933
	(0.0661)	(0.0764)	(0.0883)	(0.0644)	(0.0648)	(0.0620)
Public Hospital Available (1 = yes)			0.0566			−0.0504
			(0.120)			(0.126)
Private Hospital Available (1 = yes)			0.172			0.154*
			(0.105)			(0.0929)
Public Health Centre Available (1 = yes)			−0.0731			−0.0120
			(0.0680)			(0.0800)
Private Health Centre Available (1 = yes)			−0.0478			−0.140
			(0.104)			(0.0986)
Community Education			0.0379**			0.0435**
			(0.0180)			(0.0192)
Community Entertainment Expenditure			−0.00176			−0.00406**
			(0.00136)			(0.00166)
Mean Adults in Community			−0.0994**			−0.0755*
			(0.0422)			(0.0390)
Constant	−1.662***	−1.694***	−1.604***	−2.036***	−3.204***	−2.743***
	(0.152)	(0.149)	(0.265)	(0.217)	(0.539)	(0.584)
Endogeneity test ($p$-value)	-	0.0499	0.0590	-	0.0374	0.0584
Overidentification test ($p$-value)	-	0.4851	0.3368	-	0.3904	0.3742
Weak identification test (Cragg-Donald Wald $F$-stat)	-	66.182	65.326	-	31.830	28.812
Underidentification test ($p$-value)	-	0.000	0.000	-	0.000	0.000
Observations	2,589	2,589	2,465	2,676	2,676	2,540

*** p<0.01, ** p<0.05, * p<0.1

### Instrument variables diagnostics

For each of the regressions we cluster at the community level to partial out heterogeneity from community differences. The Pagan-Hall heteroskedasticity tests fail to reject homoscedasticity for each of the four 2SLS regressions, leading us reason to not report the GMM estimators. The IV diagnostics for the 2SLS regressions are reported as a part of Table 2.

The instruments used for 2SLS regressions with the per capita consumption expenditure measure are seen to be strong from the weak identification tests, with a Cragg-Donald Wald F-stat of 66.18 for the model without community variables (column 2), and 65.33 for the model with community variables (column 3). Comparable Stock-Yogo critical value at 5 % relative bias is 18.37, and our F-stat is well over the value. The same instruments are used for 2SLS regressions with the wealth index measure and are also seen to be strong from the weak identification tests, with a Cragg-Donald Wald F-stat of 31.83 for the model without community variables (column 5), and 28.81 for the model with community variables (column 6), compared to the same Stock-Yogo critical value at 5 % relative bias of 18.37.

The underidentification tests in all four 2SLS regressions reject the null hypothesis indicating that the reduced form coefficient matrix is full rank and the instruments are able to identify the model, satisfying the relevance assumption. The overidentification tests are all insignificant well over the alpha threshold, indicating that, assuming that at least one of the two instruments is uncorrelated with the error.

### Estimations of household economic standing

For both the per capita consumption expenditure and wealth index, the OLS underestimates the effect and is biased towards zero. The backward effect thus seem to run from poor child health creating an incentive for the household to earn more, for pursuing treatment purposes, resulting in a positive impact on household income. Using instruments we purge the effect of income on child health off the backward effect, leading to a proper estimation of the income effect alone. The instruments also purge the endogeneity stemming from any measurement bias.

The 2SLS estimate on per capita consumption expenditure is found to be more than twice as large as the OLS estimate. The significance of the Hausman-Wu test indicates that the 2SLS and OLS results are indeed statistically different from each other. Once the community variables are added into the model, the effect falls slightly but still remains over twice the OLS estimation; an increase in household per capita consumption expenditure by 1,000 rupees increase child health by 0.410 standard deviations in terms of stunting.

Similarly, the 2SLS estimate on wealth index is found to be larger than the OLS estimate, but this time, more than thrice as large. As before, once the community variables are added into the model, the effect falls slightly; the overall effect however is much larger than per capita consumption expenditure with an increase in wealth index by one unit increasing child health by 2.38 standard deviations in terms of stunting. This positive effect of income on child health in a developing country context provides further supportive evidence to a cohort of similar studies [22, 30, 57].

Per capita consumption expenditure is a short-run measure of household economic standing and has transitory components over time. The wealth index on the other hand is a more permanent measure of a household’s economic standing. Smith and Kington [58] note that income (or expenditure) in a single year does not adequately reflect the financial resources available to a household to exemplify decisions that affect health. Given that stunting is a measure of long term child health, expectedly we find a much larger effect of the wealth index on child health compared to that of per capita consumption expenditure. This simply reiterates that a household with higher wealth can provide more assurance to providing better child health compared to a household with simply higher household per capita consumption expenditure.

Under the assumption we do not have measurement bias and that our understanding of the backward effect of poor child health creating an incentive for the households to earn more is correct, from the OLS and 2SLS estimation differences we can gain an understanding whether this triggered behavioural change of earning more income is only short-term or also a long-term phenomenon. Compared to the OLS counterparts, given that the 2SLS estimation on expenditure is twice as large, and the same on wealth index is thrice as large, this behavioural change can be speculated to have both short-term and long-term effects on the household. To reiterate, this is only if we have endogeneity due to simultaneity bias and none from measurement error. Of course this speculation needs to be formally checked.

### Community level covariates

None of the community level variables indicating the presence of private or public hospitals or health centres are significant (except for private hospitals in the wealth index model, column 6, which is barely significant), containing large standard errors, making it difficult to draw any conclusions from them. However, average community level education is significant with a positive effect on child health, a finding consistent with several previous studies [47, 50]. An increase in the average community education by five years would increase child health by 0.190 standard deviations for the consumption expenditure model and by 0.218 standard deviations for the wealth index model, suggesting a positive peer effect of the locale.

High community level entertainment expenditures also carry a negative significant effect on child health, however only for the wealth index model. The reason a community level variable was constructed, instead of simply at an individual level, was to capture the peer pressure of a community that prioritizes entertainment spending. This has been built on Banerjee and Duflo’s [10] argument that poor people tend to spend a large portion of their income on entertainment which is a major constraint on their expenditure on other important daily needs. The significant coefficient in the wealth index model (column 6) provides evidence that such high entertainment expenditures do affect child health negatively by diverting available resources. An increase in community level entertainment expenditure by a hundred rupees decreases child health by 0.406 standard deviations, as per the wealth index 2SLS model.

Finally, while the number of adults in a household does not significantly affect child health, it seems an increase in the average number of adults in the community decrease child health by at least −0.0755 standard deviations in the wealth index model (column 6). However, whether this variable reflects the improved overall environmental conditions or is a direct reflection of adults helping children, is a matter that remains vague. In order to make such a differentiation, controls for the community environment conditions would have to be included, which unfortunately is not available in the dataset.

### Other covariates

Expectedly, stunting falls with age, similar to Pal [18], however we do not find significant differences between male and female children even though the coefficient indicates better health of the female child compared to the male child. Interestingly, being enrolled in school significantly positively affects child health, a result consistent in several of the models. As with many earlier studies, mothers with higher education take better care of child health while the same effect is not significant with fathers’ higher education [32–34]. Mothers with higher education, on average, increase child health by 0.305 standard deviations compared to mothers with no education.

The genetic transmission of health is significant throughout the six models; mothers who are taller by a hundred centimetres transit better child health of 0.2 standard deviations on average to their children. Having a smoker in the household is found to have significant negative effect on child health of a magnitude of −0.137 standard deviations. While this estimate is significant when controlling for per capita consumption expenditure model, it is no longer significant when controlling for the wealth index using 2SLS. Households facing shocks, expectedly, are similarly seen to have a significant negative effect on child health of a magnitude of −0.162 standard deviations.

## Conclusion

This study uses instrument variables to estimate the effect of household economic standing on child health using measures of per capita consumption expenditure and wealth index, controlling for confounders and adjusting for endogeneity. Both measures are underestimated using OLS. Once corrected using 2SLS, coefficients show that the underestimation bias is lower for the short run consumption expenditure measure compared to the long run wealth index measure.

Several important conclusions can be drawn from this. The measure used to indicate household wealth status significantly affects the estimation and should be carefully noted, especially in comparative studies. Furthermore, without using instruments we find a much smaller impact of income on child health, which is corrected when using properly estimation strategies. Does this mean that income is the most important socio economic factor, over maternal education, contributing to improvements in child health? That is a more difficult question to answer. Several studies, including Grossman [16], have showed the importance played by parental education towards better child health and while there is also a number of studies indicating that the effect of income is small, measures of permanent income or wealth on child stunting is found to be quite large and significant in this study. Thus for long run improvements in stunting, it seems household wealth remains a significant determinant.

While Meer, Miller and Rosen [59] have shown using instrument variables estimation that there is no short-run effect of wealth on health for the adult, the same is not true in the case of the child; there is significant positive effect of short-run per capita consumption expenditure on child health, albeit the effect being comparatively small.

The backward effect running from child health to household income is understood to be an inverse relationship; poor child health creates an incentive for the households to earn more income for the used sample in the study. However, an exact estimation is still needed to be done. Finally, the importance of community level factors in understanding child health is re-emphasized and that leaving them out of econometric models could lead to omitted variable bias. This study finds that while better overall community education can improve child health, increased community entertainment expenditure can also lead to deterioration in child health. Both of these factors operate through community peer effects indicating that child health is not only a household function to consider.

The findings of this study are thus suggestive that while policies catered towards improving household wealth will decrease child stunting in the long run, maternal education and the community plays an equally reinforcing role in improving child health and maybe faster routes to achieving the goal of better child health in the short run. This study stresses the importance of tackling underlying endogeneity problems when conducting such investigations in future research, and hence a stronger burden of proof should always be met before establishing, and especially quantifying, any form of such causal relationships.
